# Ultimate Intrinsic SNR in the Torso of Realistic Body Models

**DOI:** 10.1002/mrm.70202

**Published:** 2025-11-26

**Authors:** Yuting Wang, Markus W. May, Marcel Gratz, Mark E. Ladd, Stephan Orzada

**Affiliations:** ^1^ Medical Physics in Radiology German Cancer Research Center (DKFZ) Heidelberg Germany; ^2^ Faculty of Physics and Astronomy Heidelberg University Heidelberg Germany; ^3^ High‐Field and Hybrid MR Imaging University Hospital Essen Essen Germany; ^4^ Erwin L. Hahn Institute for MRI University Duisburg‐Essen Essen Germany; ^5^ Faculty of Medicine Heidelberg University Heidelberg Germany

**Keywords:** in silico, MRI, torso, uiSNR

## Abstract

**Purpose:**

This work aims to investigate how the ultimate intrinsic signal‐to‐noise ratio (uiSNR) varies with increasing static magnetic field $B_{0}$ in the torso of realistic body models.

**Methods:**

A dipole cloud was positioned around the realistic body model and randomly excited. The volume integral solver MARIE was used to calculate the corresponding electromagnetic fields. The uiSNR maps were calculated using these electromagnetic bases and were fitted with the power law for different $B_{0}$ ranges.

**Results:**

The uiSNR could be reliably calculated in regions deeper than 3 cm, where convergence of uiSNR over the number of basis vectors was achieved. In a lower magnetic field range (from 0.55 to 3 T), the uiSNR increases roughly linearly versus $B_{0}$ with small variation throughout the torso (Ella: uiSNR ∝ *B*_0_^0.96±0.07^, Duke: uiSNR ∝ *B*_0_^0.98±0.10^). In an upper magnetic field range (from 5 to 14 T), the uiSNR increases superlinearly in the torso (Ella: uiSNR ∝ *B*_0_^1.86±0.25^, Duke: uiSNR ∝ *B*_0_^1.99±0.28^), with a larger variation correlated to the heterogeneous structure of the body model.

**Conclusion:**

The superlinear scaling exponent in the upper magnetic field range indicates the promise of applying UHF MRI for body imaging.

## 1 Introduction

Ultra‐high field (UHF) MRI with a magnetic field strength of *B*_0_ ≥ 7 T has the potential to open new opportunities for diagnostics and research in human health and physiology [1, 2]. The enhanced polarization at higher $B_{0}$ indicates an improved signal‐to‐noise ratio (SNR), which is desirable since a higher SNR can be used either to improve imaging resolution or to shorten scan time. It also enables the more practical measurement ofX‐nuclei and facilitates applications in spectroscopy and CEST imaging.

Although it is well established that SNR increases with $B_{0}$, the specific scaling behavior is influenced by factors such as the RF system and the object or subject being imaged [3], making it a complex issue that requires further study. The concept of ultimate intrinsic SNR (uiSNR) [4, 5, 6] provides an insightful simplification by excluding the coil noise [7]. It considers the intrinsic thermal noise from the sample only and is defined as the maximum intrinsic SNR that is achievable by any RF coil configuration. For a sample, the uiSNR is theoretically an electromagnetic limit dictated by Maxwell's equations that is independent of specific receive array designs and can therefore be used as a performance metric for receive arrays [8, 9, 10, 11, 12].

The uiSNR problem in MRI has been investigated in different ways. For objects with homogeneous properties and simple geometry, analytical and semi‐analytical research has predicted an approximately linear growth of uiSNR for low $B_{0}$ followed by a transition toward a superlinear increase versus $B_{0}$ for deep positions in the tissue at higher field strengths [6, 13, 14, 15]. Realistic simulation works have confirmed the superlinear increase at high $B_{0}$ in the center part of realistic head models [16, 17, 18]. The tendency toward a better‐than‐linear increase has also been verified by experiments measuring either a spherical phantom or a human head [3, 19, 20].

However, restricted by heavy computational burden due to the larger problem size, the study of uiSNR in the torso region has remained limited. Nevertheless, improved SNR in torso imaging may offer potential benefits for a range of clinical applications. In this work, we focused on the torso region of realistic body models to investigate uiSNR as a function of $B_{0}$, using electromagnetic simulations based on realistic body models. Preliminary results of this work were presented at the 2025 ISMRM annual meeting [21].

## 2 Methods

### 2.1 Realistic Body Models

The Duke and the Ella body models from the Virtual Family [22, 23] (IT'IS Foundation, Zürich, Switzerland) were used for the uiSNR simulations. These body models were exported as voxel models with 3 mm isotropic resolution with various types of tissues, which serve as a straightforward and practical segmentation of organs in the torso.

The full Duke model contains 2 538 815 non‐air voxels in a 182 × 96 × 603 voxel grid. To reduce the computational burden, it was truncated from the neck to the proximal third of the femur, containing the region of interest for torso imaging. The truncated model contains 1 841 688 non‐air voxels of 48 tissue types in a 180 × 75 × 266 voxel grid. The Ella model contains 2 085 940 non‐air voxels of 52 tissue types in a 169 × 95 × 552 voxel grid. The smaller size of Ella enables simulations with the complete body model given the computational resources available to us.

**FIGURE 1:**
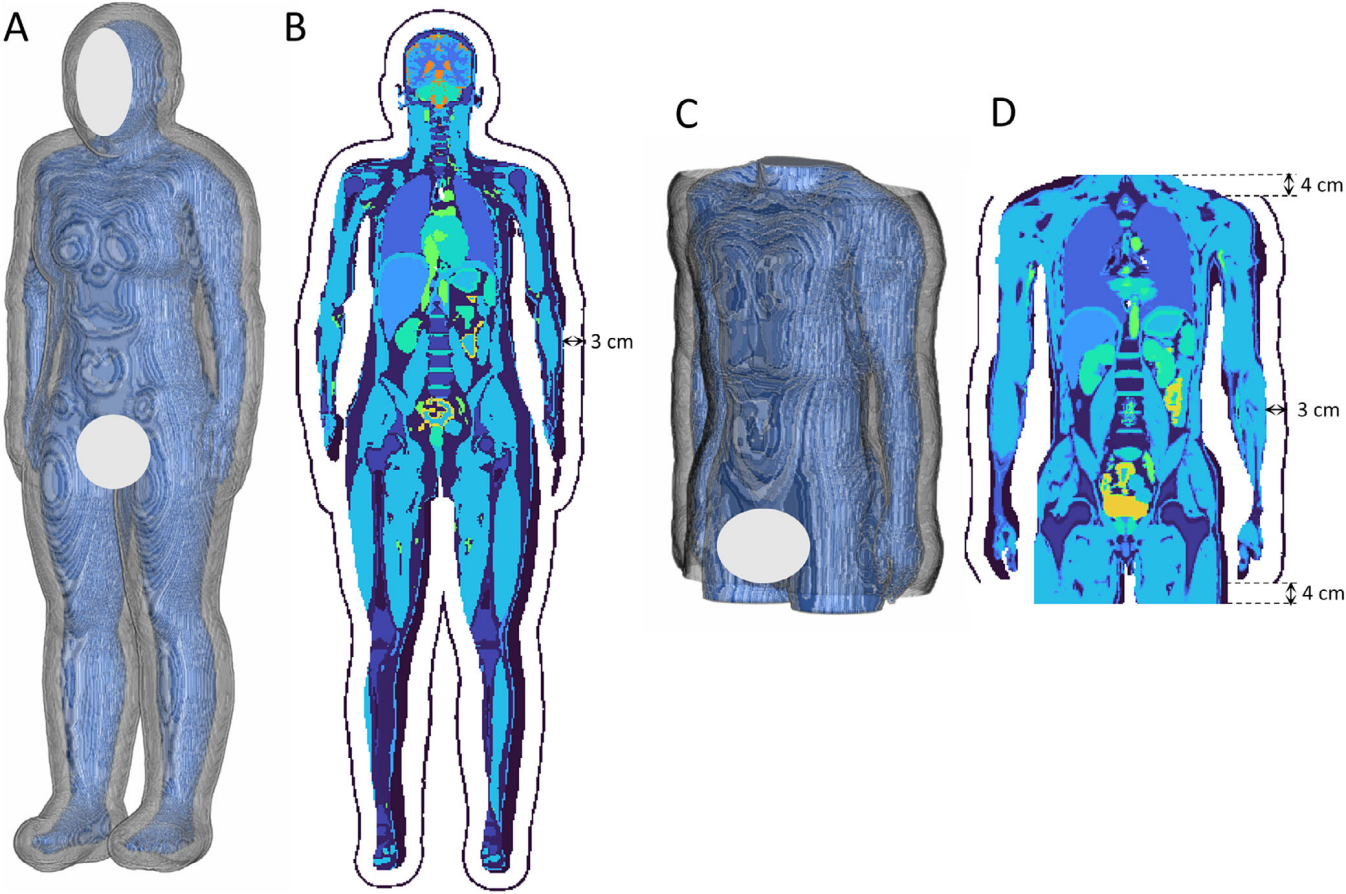
(A) 3D view of the Ella model and the voxelized dipole cloud conformal to the body surface. (B) A coronal cross section of the Ella model with the dipole cloud. (C) 3D view of the truncated Duke model and the voxelized dipole cloud conformal to the body surface. (D) A coronal cross section of the truncated Duke model with the dipole cloud.

Simulations were performed at field strengths of 0.55, 1.5, 3, 5, 7, 10.5, 11.7, and 14 T. At each field strength, frequency‐dependent values of permittivity and conductivity [24] were assigned to different tissues in the body models (Figure 1).

### 2.2 Electromagnetic Bases

The calculation of the uiSNR requires a complete set of solutions to Maxwell's equations in the sample. The method proposed by Guerin et al. [16, 25] was followed to set up a voxelized dipole cloud and to compute a random basis set of electromagnetic fields for each field strength, which increasingly approximates a complete basis set as the number of electromagnetic bases grows.

The voxel grid containing the body model was expanded to include a shell‐shaped region for dipole placement. The centers of the dipole voxels are located between 3.0 and 3.5 cm from the body surface, forming a 5 mm‐thick dipole cloud that serves as the basis support for reproducing the electromagnetic fields generated by external RF sources. Three electric and three magnetic dipoles, oriented along the three Cartesian axes, were placed at the center of each dipole voxel to enable the use of Fourier operators in the fast electromagnetic solver MARIE [26, 27, 28].

For the smaller Ella model, simulations were performed using the complete body model together with a fully enclosing dipole cloud, in accordance with the definition of uiSNR. For the larger Duke model, a truncated model was employed. In this case, a fully encompassing dipole cloud would cause strong boundary artifacts and non‐physical overestimation near the truncation cross sections. To avoid this, we used a dipole cloud arranged circumferentially around the truncated Duke model, 4 cm shorter than the body model on both ends. The resulting SNR corresponds to the optimal SNR [18] of the truncated body model, which provides a good approximation to the uiSNR of the full body model specifically in the torso region. Validation of this approximation is presented in the Supporting Information. For clarity and consistency, this approximation is referred to also as uiSNR throughout the manuscript. The body models and their corresponding dipole cloud setups are illustrated in Figure 1.

The size of each basis set was limited to 3000 random electromagnetic bases, as a practical compromise between computational feasibility and simulation accuracy. These basis sets were computed in MATLAB R2023b (MathWorks, Natick, MA, USA) on a server equipped with Ubuntu 24.04 LTS featuring 1.5 TB RAM, dual AMD Epyc 9654 processors (each with 96 cores/192 threads), and two NVIDIA H100 GPUs (each with 80 GB memory). Two GPUs were used in parallel to accelerate the FFT‐based operations implemented in MARIE and the solving of linear systems using MATLAB's built‐in BICGSTAB solver.

### 2.3 Ultimate Intrinsic SNR Calculation

From the electromagnetic basis set described above, the uiSNR at position r can be expressed as [6, 8]

$$\text{uiSNR}\left(r\right)=\frac{{\omega M}_{0}\left(r\right)\Delta V}{\sqrt{4k_{b}T\Delta f}}\sqrt{{B_{1}^{-}}^{H}\left(r\right)\Psi^{-1}B_{1}^{-}\left(r\right)},$$

where ω is the Larmor frequency, $M_{0}$ is the equilibrium magnetization, ΔV is the volume of a voxel, $k_{b}$ is Boltzmann's constant, T is the absolute temperature, Δf is the receive bandwidth, $\Psi_{\mathrm{ij}}=\int_{V}\sigma \left(r^{\prime }\right)E_{i}\left(r^{\prime }\right)E_{j}^{H}\left(r^{\prime }\right)d^{3}r^{\prime }$ is the noise covariance matrix and $B_{1}^{-}\left(r\right)$ is the vector of RF receive fields. The complete basis set was employed for the computation of the uiSNR.

The analysis of uiSNR scaling with $B_{0}$ only needs to focus on the *B*_0_‐dependent part of this expression. The term $\sqrt{{B_{1}^{-}}^{H}\left(r\right)\Psi^{-1}B_{1}^{-}\left(r\right)}$ involves the spatial distribution of the RF fields and is RF frequency‐dependent. Besides, both the Larmor frequency term $\omega =\gamma B_{0}$ and the equilibrium magnetization term $M_{0}\left(r\right)=\rho \left(r\right)\frac{\gamma^{2}\hbar^{2}}{4k_{b}T}\;B_{0}$ (for spin 1/2 ^1^H) are proportional to $B_{0}$, giving an extra $B_{0}^{2}$ dependence. Thus, for our purposes, it is sufficient to consider the term $B_{0}^{2}\sqrt{{B_{1}^{-}}^{H}\left(r\right)\Psi^{-1}B_{1}^{-}\left(r\right)}$, to which the uiSNR is proportional.

It is worth noting that, among the omitted terms, the proton density ρ(r) from the magnetization $M_{0}\left(r\right)$ also has a spatial dependence. When the model used in simulations is heterogeneous, the uiSNR will vary throughout the model due to the non‐uniform distribution of proton density. In this work, due to the absence of an available proton density database, spatial uiSNR variations resulting from this term are not considered.

### 2.4 Power Law Fitting of uiSNR

After obtaining the uiSNR maps for field strengths from 0.55 to 14 T, the uiSNR was fitted for each position with the power law:

$$\text{uiSNR}\left(r\right)\propto B_{0}^{\alpha (r)}.$$

In some previous studies [17, 19], the SNR data were fitted with a single power‐law exponent across the entire range of $B_{0}$. However, this approach does not adequately capture the uiSNR behavior observed in our study. Improved fitting is achieved by separating the field strengths into a low‐field regime (0.55–3 T) and a high‐field regime (5–14 T). Piecewise power law fittings were applied using the Levenberg–Marquardt algorithm with a tolerance of 1e‐10 in MATLAB.

## 3 Results

### 3.1 Convergence With Position Dependence

**FIGURE 2:**
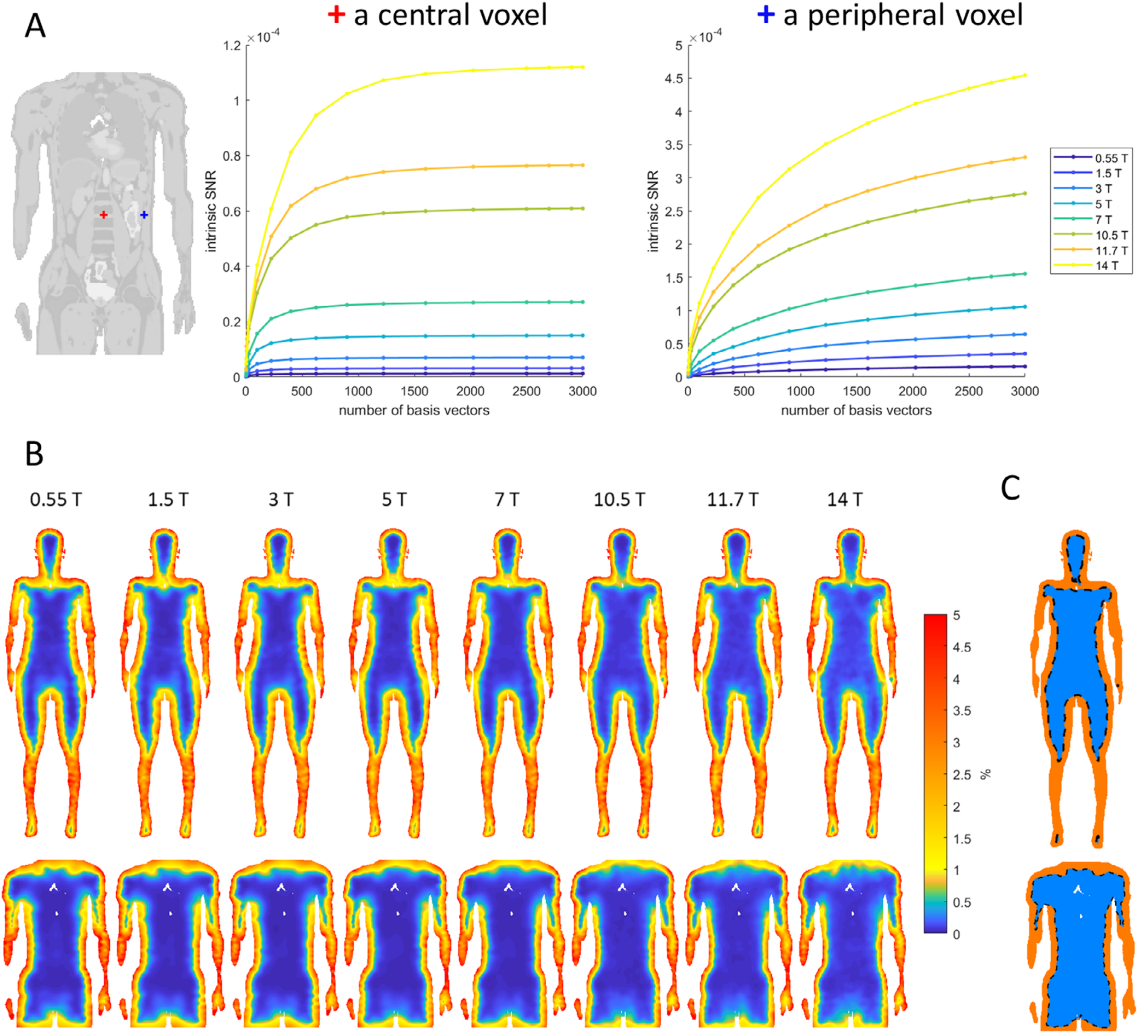
(A) Intrinsic SNR versus number of electromagnetic basis vectors in a central voxel and a peripheral voxel in the truncated Duke model. (B) Percent SNR change from 2900 to 3000 electromagnetic bases for field strengths from 0.55 to 14 T in the Ella model (top) and the truncated Duke model (bottom). (C) The masks of convergence (green) and their boundaries (dashed lines) in the Ella model (top) and the truncated Duke model (bottom). Air‐filled regions (such as the trachea lumen and the esophagus lumen) are not covered by the mask and thus appear blank in the images.

The approximation to uiSNR improves with increasing basis set size, though the rate of convergence differs depending on the position within the body model. Convergence is reached rapidly in deeper regions of the body model, whereas in peripheral voxels, the SNR does not plateau with 3000 basis vectors and is still sub‐optimal (Figure 2A).

Figure 2B shows percent SNR change when increasing the number of basis vectors from 2900 to 3000. As a practical compromise, we defined a convergence criterion such that the relative change remains below 1% for all simulated field strengths. Voxels satisfying this criterion formed a mask with convergence for each body model, as illustrated in Figure 2C.

### 3.2 uiSNR Maps

**FIGURE 3:**
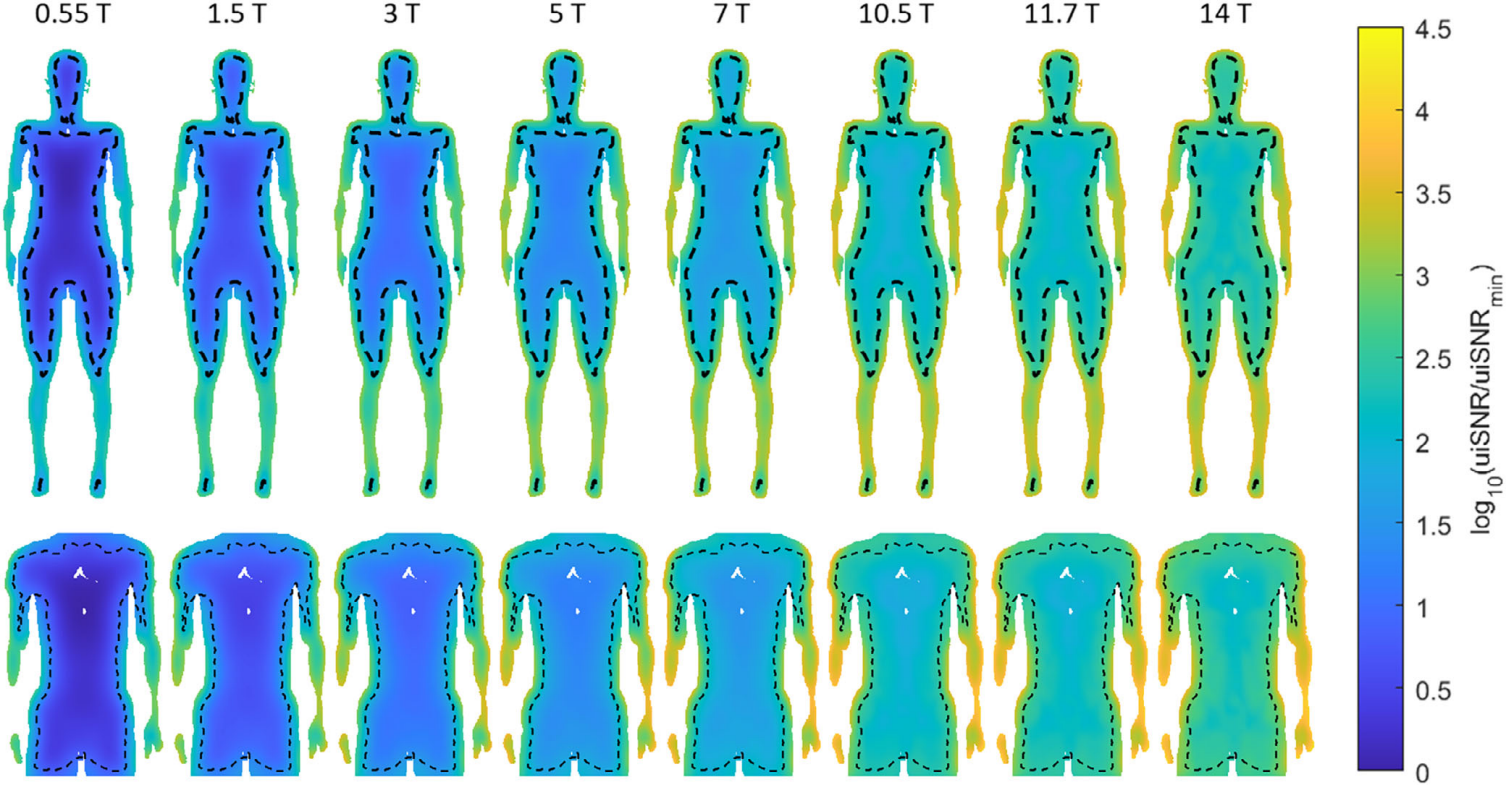
Logarithmic uiSNR maps in the Ella model and the truncated Duke model in coronal view for field strengths from 0.55 to 14 T. The uiSNR values are normalized to the uiSNR of the voxel with the lowest value at 0.55 T, denoted as uiSNR_min_. The maps are not weighted by the proton density distribution and are therefore not a representation of the realistic uiSNR distribution. Black dashed lines indicate the boundary of the mask of convergence.

Figure 3 presents maps of the *B*_0_‐dependent component of uiSNR in a coronal cross section of the body models at various field strengths, shown on a logarithmic scale. The boundary of the mask with convergence is marked by the dashed line, which is roughly 3 cm beneath the body surface. In the center of the torso, uiSNR increases by nearly two orders of magnitude from 0.55 to 14 T. In the peripheral region, although the SNR values with the available basis sets are still sub‐ultimate due to lack of convergence, they are about an order of magnitude higher than the central uiSNR.

### 3.3 uiSNR Scaling Exponent

**FIGURE 4:**
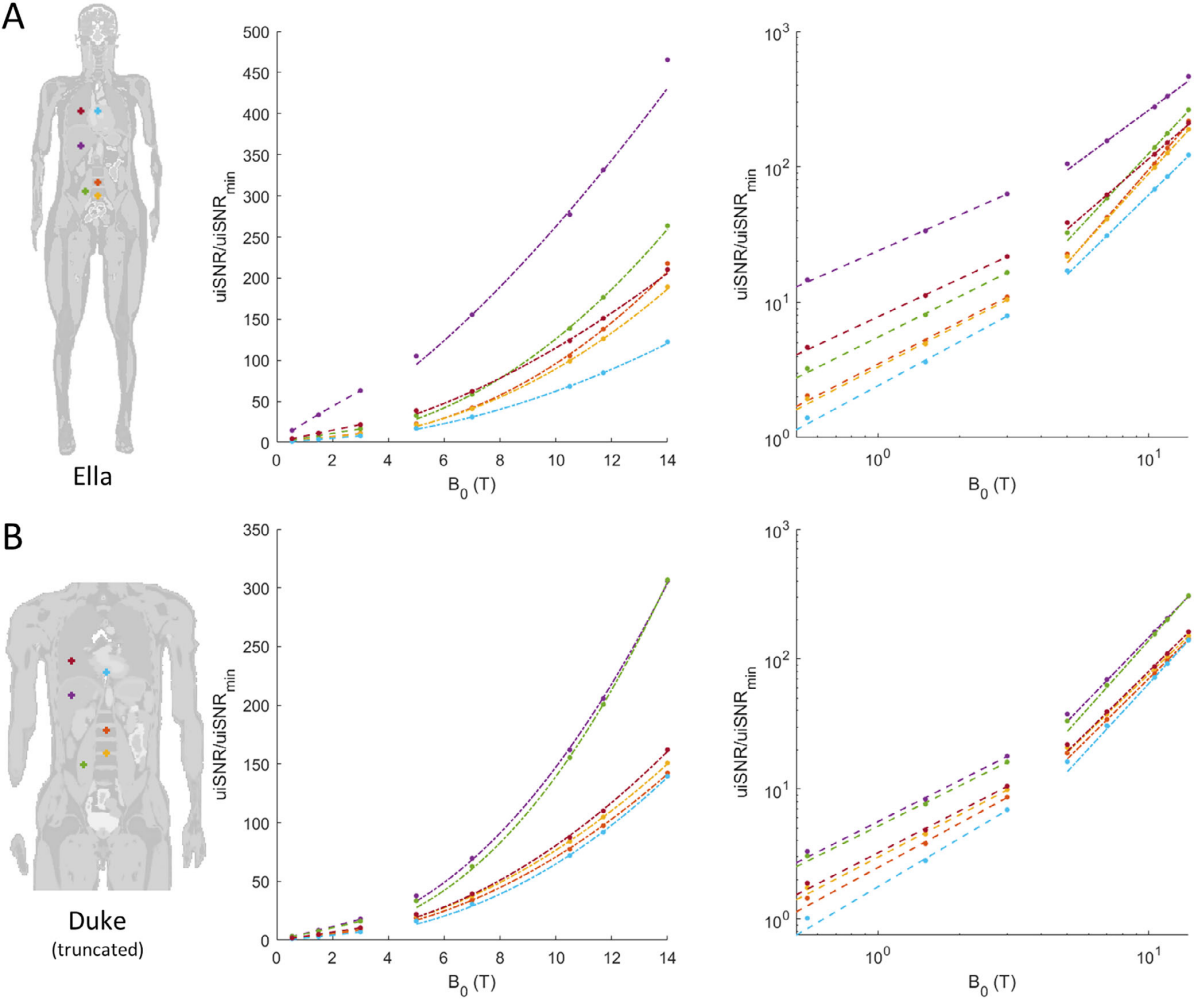
uiSNR (normalized to the uiSNR of the voxel with the minimum value at 0.55 T) versus B_0_ (for B_0_ from 0.55 to 14 T) at selected example positions in (A) the Ella model and (B) the truncated Duke model on both linear scale and double‐logarithmic scale. Markers show data points obtained from simulations. Dashed lines show the power law fitting of the data from 0.55 to 3 T. Dash‐dotted lines show the power law fitting of the data from 5 to 14 T.

Figure 4 shows the uiSNR as a function of $B_{0}$ at several selected positions in the Ella and Duke body models, plotted on both linear and logarithmic scales. In the log–log plot, the data points do not follow a straight line, indicating that uiSNR does not scale with $B_{0}$ according to a single power law of the form ${\text{uiSNR}\left(r\right)\propto B}_{0}^{\alpha }$. Instead, the slope increases with $B_{0}$, suggesting a *B*_0_‐dependent exponent. To capture this behavior, the $B_{0}$ range was empirically divided into two regimes: 0.55–3 T (lower field) and 5–14 T (higher field). Within each regime, the slope remains approximately constant, and the uiSNR scaling can be reasonably described by a power law. This transition in scaling behavior arises from two distance regimes of RF fields, with lower fields corresponding to the near‐field regime and higher fields to the far‐field regime [6].

**FIGURE 5:**
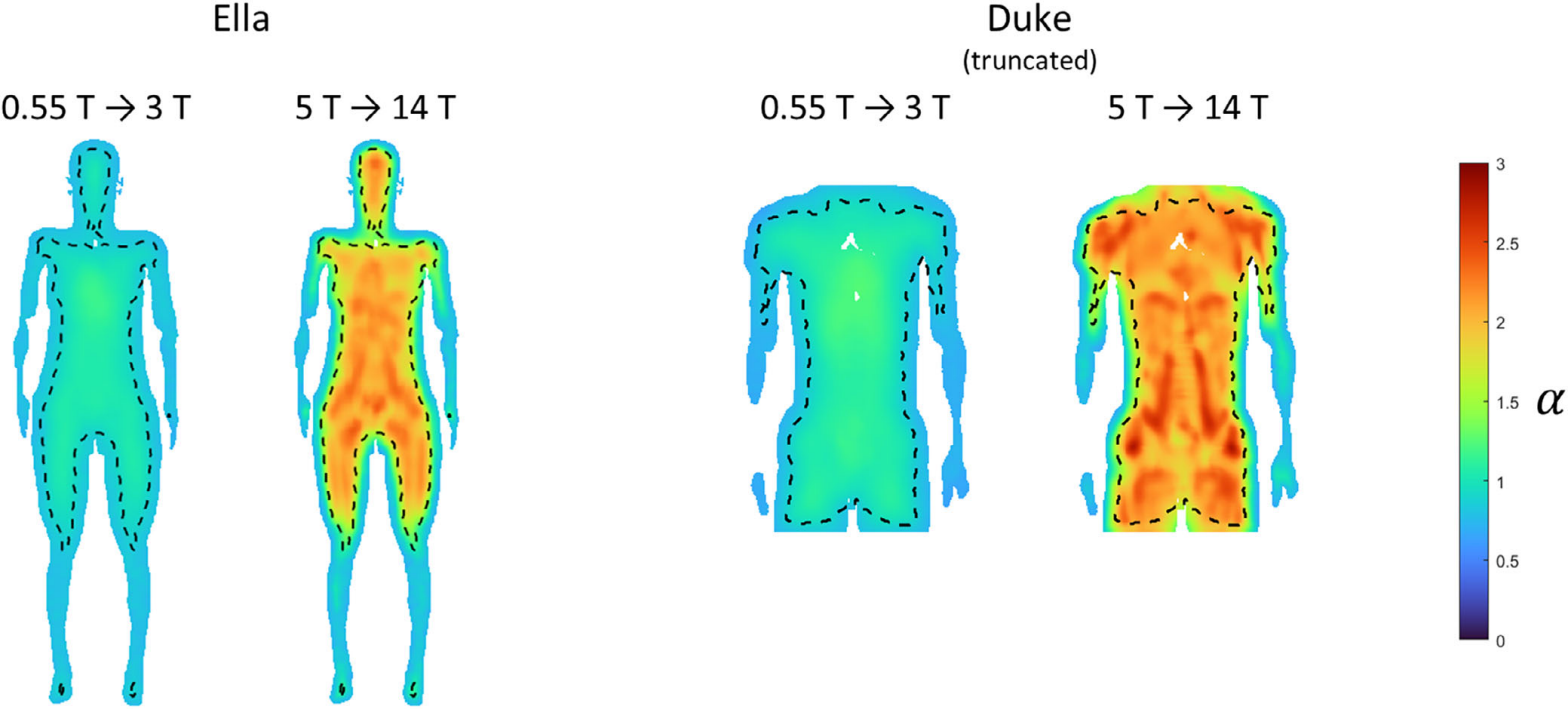
Scaling exponent maps of the uiSNR in the Ella model and the truncated Duke model for lower field strengths from 0.55 to 3 T and upper field strengths from 5 to 14 T. Black dashed lines indicate the boundaries of the region where convergence toward uiSNR is achieved.

Figure 5 shows the uiSNR power‐law scaling exponent maps for the lower field range and the upper field range. In the lower field range, the scaling exponent is more even throughout the torso, with values of 0.96 ± 0.07 for Ella and 0.98 ± 0.10 for Duke. In the upper field range, the increase exponent varies more and vaguely reveals the anatomical structure of the body models. The exponent in the upper field range is greater than in the lower field range, with values of 1.86 ± 0.25 for Ella and 1.99 ± 0.28 for Duke in the torso. Table 1 shows statistics of the uiSNR scaling exponent within the mask of convergence for major organs and tissues in Ella and Duke. For many organs of interest, including the liver, uterus and prostate, the scaling exponent is around 1 at lower field strengths, suggesting a near‐linear increase, and approaches a value around 2 at higher fields, indicating the increase is roughly quadratic. At high field strengths, the scaling exponents of Ella's organs tend to be lower than those of the corresponding organs in Duke. For the Ella model, the head part was also included in the simulation, and the scaling exponents are comparable with those obtained in previous studies [17, 19].

**TABLE 1:** uiSNR scaling exponents for major tissues and organs in the Ella model and the truncated Duke model.

Tissue/organ	Scaling exponent α			
	Ella		Duke	
	0.55–3 T	5–14 T	0.55–3 T	5–14 T
Bone	0.95 ± 0.07	1.80 ± 0.30	0.95 ± 0.08	1.95 ± 0.30
Fat	0.94 ± 0.06	1.78 ± 0.28	0.96 ± 0.09	1.92 ± 0.28
Muscle	0.94 ± 0.05	1.83 ± 0.27	0.93 ± 0.08	1.99 ± 0.33
Liver	0.99 ± 0.07	1.95 ± 0.20	1.00 ± 0.10	2.02 ± 0.24
Lung	0.96 ± 0.05	1.86 ± 0.19	1.00 ± 0.08	2.03 ± 0.21
Heart muscle	1.03 ± 0.09	1.95 ± 0.21	1.06 ± 0.12	2.00 ± 0.19
Kidney (cortex)	0.99 ± 0.05	1.97 ± 0.21	1.06 ± 0.08	2.12 ± 0.15
Kidney (medulla)	0.99 ± 0.04	2.08 ± 0.15	1.06 ± 0.07	2.18 ± 0.12
Pancreas	0.97 ± 0.04	1.98 ± 0.12	1.10 ± 0.06	2.26 ± 0.12
Spleen	0.96 ± 0.05	1.94 ± 0.19	0.96 ± 0.05	2.05 ± 0.20
Stomach	1.00 ± 0.08	2.01 ± 0.24	1.00 ± 0.11	2.06 ± 0.26
Ovary	1.04 ± 0.02	2.05 ± 0.05	—	—
Uterus	1.02 ± 0.04	2.27 ± 0.16	—	—
Prostate	—	—	1.10 ± 0.02	2.04 ± 0.13
Torso	**0.96 ± 0.07**	**1.86 ± 0.25**	**0.98 ± 0.10**	**1.99 ± 0.28**
Gray matter	0.89 ± 0.05	1.83 ± 0.26	—	—
White matter	0.88 ± 0.04	1.80 ± 0.25	—	—
CSF	0.95 ± 0.06	1.97 ± 0.20	—	—
Head	**0.89 ± 0.05**	**1.79 ± 0.25**	—	—
Whole body	**0.95 ± 0.06**	**1.84 ± 0.27**	—	—

*Note*: Bold values indicate evaluations at broader anatomical levels (torso, head, and whole body).

**FIGURE 6:**
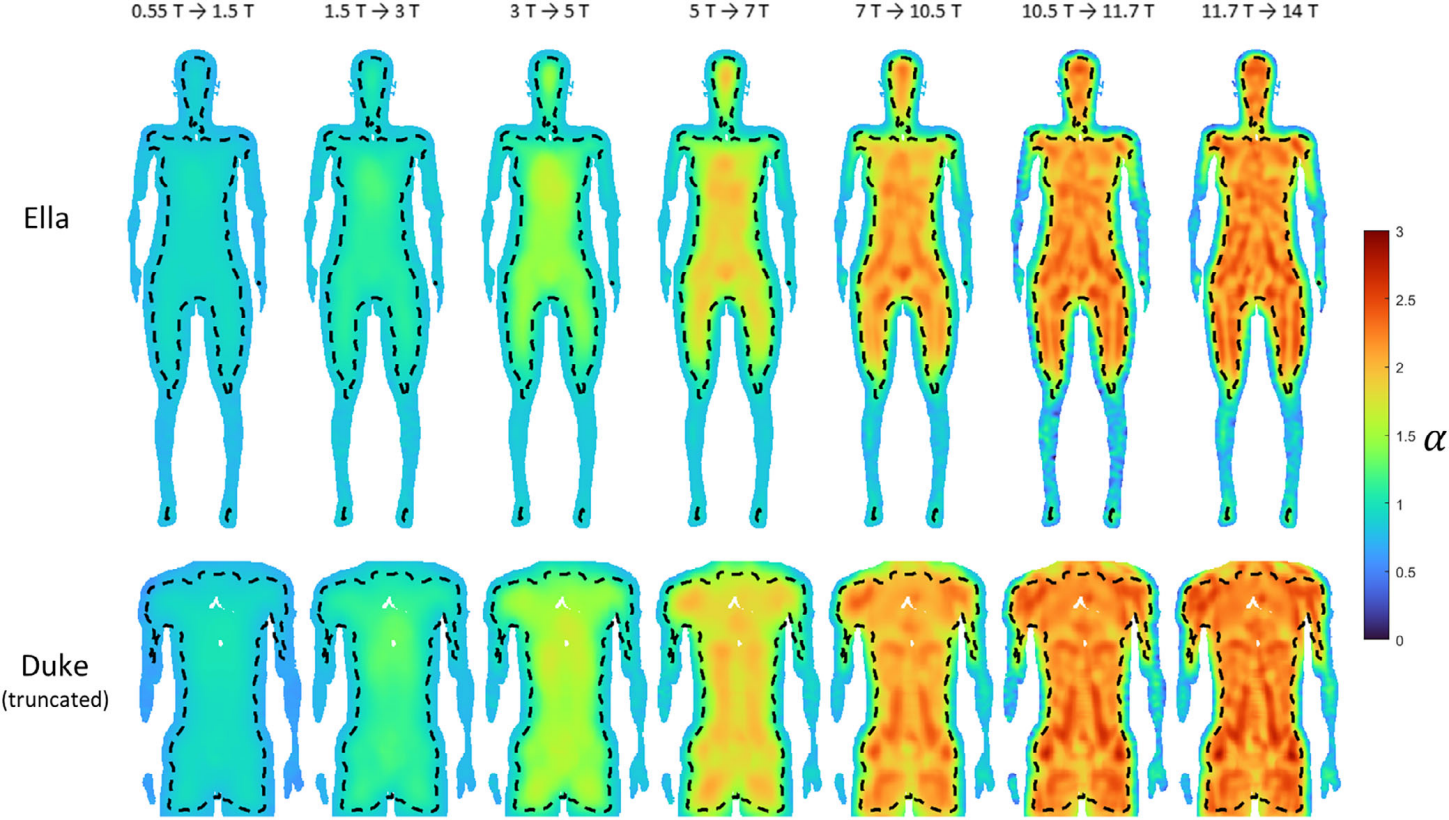
Effective scaling exponent maps in the Ella model and the truncated Duke model, calculated between adjacent field strengths.

The uiSNR increase factors between adjacent field strengths and the corresponding effective scaling exponents (calculated as $\log\left(\text{uiSN}R^{\text{high}}/\text{uiSN}R^{\mathrm{low}}\right)/\log\left(B_{0}^{\text{high}}/B_{0}^{\mathrm{low}}\right)$) are shown in Table 2 (Ella) and Table 3 (Duke). The spatial distribution of the effective scaling exponent is shown in Figure 6, in which a transition from approximately linear to superlinear uiSNR growth can be observed.

**TABLE 2:** uiSNR increase factors and corresponding effective scaling exponents (mean within each tissue/organ) for major tissues and organs in the Ella model, calculated between adjacent field strengths.

Tissue/organ	0.55–1.5 T		1.5–3 T		3–5 T		5–7 T		7–10.5 T		10.5–11.7 T		11.7–14 T	
	Factor	Exp	Factor	Exp	Factor	Exp	Factor	Exp	Factor	Exp	Factor	Exp	Factor	Exp
Bone	2.42	0.88	1.98	0.98	1.83	1.17	1.63	1.44	2.05	1.76	1.24	1.96	1.45	2.06
Fat	2.42	0.88	1.96	0.97	1.82	1.16	1.62	1.43	2.04	1.74	1.23	1.94	1.44	2.01
Muscle	2.41	0.88	1.96	0.97	1.80	1.14	1.62	1.42	2.07	1.78	1.25	2.03	1.47	2.14
Liver	2.47	0.90	2.05	1.03	1.95	1.30	1.73	1.61	2.18	1.91	1.25	2.08	1.47	2.15
Lung	2.43	0.88	1.99	0.99	1.85	1.20	1.66	1.50	2.10	1.82	1.24	2.02	1.46	2.10
Heart muscle	2.52	0.92	2.12	1.08	2.05	1.39	1.77	1.69	2.19	1.93	1.25	2.05	1.46	2.10
Kidney (cortex)	2.48	0.90	2.03	1.02	1.92	1.27	1.73	1.62	2.21	1.95	1.25	2.08	1.46	2.10
Kidney (medulla)	2.49	0.91	2.04	1.03	1.94	1.30	1.76	1.68	2.30	2.05	1.27	2.20	1.49	2.22
Pancreas	2.43	0.89	2.01	1.01	1.90	1.25	1.71	1.59	2.20	1.94	1.26	2.12	1.48	2.19
Spleen	2.43	0.88	2.00	1.00	1.87	1.22	1.68	1.54	2.16	1.90	1.26	2.10	1.48	2.17
Stomach	2.47	0.90	2.05	1.04	1.95	1.30	1.74	1.64	2.23	1.97	1.26	2.16	1.49	2.21
Ovary	2.54	0.93	2.13	1.09	2.08	1.43	1.85	1.83	2.30	2.06	1.25	2.05	1.43	1.98
Uterus	2.52	0.92	2.09	1.06	2.05	1.40	1.87	1.85	2.49	2.24	1.29	2.38	1.53	2.38
Torso	**2.43**	**0.89**	**1.99**	**0.99**	**1.86**	**1.20**	**1.66**	**1.50**	**2.11**	**1.82**	**1.25**	**2.03**	**1.46**	**2.11**
Gray matter	2.27	0.82	1.89	0.92	1.77	1.11	1.62	1.41	2.07	1.78	1.25	2.04	1.48	2.17
White matter	2.26	0.81	1.88	0.91	1.74	1.09	1.59	1.37	2.04	1.74	1.24	2.02	1.47	2.15
CSF	2.38	0.86	1.98	0.98	1.88	1.23	1.71	1.58	2.19	1.92	1.26	2.14	1.49	2.22
Head	**2.29**	**0.83**	**1.90**	**0.93**	**1.76**	**1.10**	**1.60**	**1.38**	**2.04**	**1.74**	**1.24**	**2.00**	**1.46**	**2.12**
Whole body	**2.42**	**0.88**	**1.98**	**0.98**	**1.83**	**1.18**	**1.64**	**1.46**	**2.08**	**1.79**	**1.24**	**2.01**	**1.46**	**2.10**

*Note*: Bold values indicate evaluations at broader anatomical levels (torso, head, and whole body).

**TABLE 3:** uiSNR increase factors and corresponding effective scaling exponents (mean within each tissue/organ) for major tissues and organs in the truncated Duke model, calculated between adjacent field strengths.

Tissue/organ	0.55–1.5 T		1.5–3 T		3–5 T		5–7 T		7–10.5 T		10.5–11.7 T		11.7–14 T	
	Factor	Exp	Factor	Exp	Factor	Exp	Factor	Exp	Factor	Exp	Factor	Exp	Factor	Exp
Bone	2.37	0.86	1.99	0.99	1.87	1.22	1.68	1.53	2.12	1.84	1.25	2.04	1.47	2.13
Fat	2.39	0.87	2.01	1.00	1.89	1.24	1.68	1.52	2.11	1.83	1.24	2.02	1.46	2.10
Muscle	2.34	0.84	1.97	0.97	1.85	1.19	1.67	1.50	2.14	1.86	1.25	2.09	1.48	2.19
Liver	2.45	0.89	2.08	1.05	1.97	1.32	1.73	1.62	2.18	1.91	1.26	2.10	1.48	2.18
Lung	2.46	0.90	2.07	1.04	1.96	1.31	1.73	1.61	2.19	1.92	1.26	2.10	1.48	2.18
Heart muscle	2.53	0.92	2.17	1.11	2.06	1.40	1.75	1.66	2.17	1.91	1.25	2.07	1.47	2.14
Kidney (cortex)	2.53	0.92	2.16	1.11	2.10	1.44	1.83	1.79	2.29	2.03	1.26	2.17	1.49	2.22
Kidney (medulla)	2.54	0.93	2.17	1.12	2.12	1.47	1.85	1.82	2.33	2.09	1.27	2.21	1.50	2.26
Pancreas	2.57	0.94	2.23	1.15	2.17	1.52	1.86	1.84	2.34	2.09	1.28	2.26	1.52	2.34
Spleen	2.38	0.86	2.00	1.00	1.88	1.23	1.68	1.54	2.17	1.91	1.26	2.14	1.49	2.21
Stomach	2.45	0.89	2.08	1.05	1.98	1.32	1.73	1.63	2.20	1.93	1.26	2.13	1.49	2.21
Prostate	2.52	0.92	2.25	1.17	2.22	1.56	1.84	1.81	2.22	1.97	1.25	2.07	1.46	2.11
Torso	**2.39**	**0.87**	**2.02**	**1.01**	**1.91**	**1.25**	**1.69**	**1.55**	**2.15**	**1.87**	**1.25**	**2.08**	**1.48**	**2.17**

*Note*: Bold values indicate evaluations of the entire torso.

## 4 Discussion

### 4.1 Limitations

Due to the limited availability of time and computational resources, several compromises were necessary. These include limiting the simulation to two body models, simulating only a few field strengths, truncating the Duke model, using a moderate voxel size of 3 mm, and restricting the number of basis vectors in each basis set to 3000.

To give the reader a sense of the computational resources involved, we provide the following estimates based on our current implementation. For each Ella model simulation with 3000 basis vectors, the simulation process may take up to 70 h. Storing the full set of basis vectors requires approximately 280 GB for the electric fields and an additional 280 GB for the magnetic fields. The peak RAM usage during computation reaches approximately 1.3 TB. Although further optimization of memory usage and computational efficiency is possible, such as reduced‐order modeling [29, 30] and tensor decomposition for volume integral equations [31, 32], this work focuses on exploring field strength and anatomical dependence of uiSNR, rather than on technical improvements of the implementation.

The Ella model and the Duke model simulated in this study represent typical physical builds of both sexes. It would be interesting to also employ body models of different habitus and postures as well as investigate the variation of uiSNR in pediatric subjects.

Field strengths were sparsely sampled in the simulated field range, making separation of different scaling regimes challenging. Additionally, the transition between the two regimes does not happen abruptly at a certain $B_{0}$. Some of the sampled field strengths may be within the transition region, which means the separation in this work does not cleanly divide the two regimes and is just an approximation.

For the basis sets containing 3000 basis vectors each in this work, convergence is not achieved in the peripheral region of the torso model. However, deep inside the body is where the uiSNR is the lowest, restricting the image quality for the target field of view. The sub‐optimal intrinsic SNR obtained in the region close to the surface, which can also be regarded as a lower bound of the uiSNR, is already much greater than the central uiSNR. We therefore do not consider the lack of convergence close to the surface to be a critical issue.

### 4.2 Comparison Between Two Body Models

The two body models represent the anatomical differences between the sexes and also exhibit distinct overall body sizes. The different organs present in the male and female pelvic cavities lead to distinct uiSNR distributions. In addition, sex‐specific anatomical differences may lead to differences in the relative positions of organs within the body, which may partly explain the differences in scaling exponents observed for the same organ across models. Moreover, as has been shown in homogeneous spheres [6, 14], sample size has an impact on [1] the transition frequency between the lower field range and the upper field range and [2] the uiSNR scaling exponents in both scenarios. In this study, the same field strength range separation was applied to both body models of different sizes, which may be another factor contributing to the differences and variations in the scaling exponents.

### 4.3 Torso Imaging at UHF

The calculated SNR increase factors between adjacent field strengths may facilitate estimation of the expected SNR gains across typical scanner upgrades. Our results imply that the rate of uiSNR improvement with increasing field strength is substantially higher at UHF. If the field strength doubles from 7 to 14 T, the torso uiSNR is expected to increase fourfold. In contrast, if the field strength is increased from 1.5 to 3 T, the uiSNR is expected to double in the torso. Our Ella simulations include both head and torso, showing similar power law exponents for uiSNR scaling with $B_{0}$ in both regions. These results show that UHF MRI has potential to provide significant gains not only in the brain but also in the torso, where improved image quality can directly benefit applications such as cancer diagnosis.

However, the advantage of substantial uiSNR increase at high $B_{0}$ may be offset by challenges at UHF, including $B_{1}^{+}$ inhomogeneity, increased power deposition, and difficulties in hardware design and construction. If these challenges can be adequately addressed, UHF body imaging may hold considerable promise, as the enhanced SNR can be exploited to improve resolution and reduce scan time.

### 4.4 Extrapolation to X‐Nuclei

Although the analysis above is performed for hydrogen MRI, the conclusion can be easily extrapolated to X‐nuclei MRI. The eight simulations in this work correspond to eight frequencies between 23 and 596 MHz, with a linear to superlinear transition frequency observed between 127.7 and 212.9 MHz. It is straightforward to convert the frequencies to corresponding fields for nuclei other than ^1^H using gyromagnetic ratios. Field ranges of different scaling patterns for several common nuclei are given in Table S1. Most nuclei other than ^1^H have a much smaller gyromagnetic ratio, resulting in a broader range of linear increase and thus a higher transition magnetic field to superlinear increase.

## 5 Conclusion

In this work, we applied electromagnetic simulations to the Ella and the Duke body models and showed that, in the center of the torso, the uiSNR increases with $B_{0}$ about linearly from 0.55 to 3 T and superlinearly from 5 to 14 T, with fitted scaling exponents of 1.86 ± 0.25 for Ella and 1.99 ± 0.28 for Duke. Although the results demonstrate consistent trends across two models, they are specific to the simulated anatomies, and should be generalized with caution. Nonetheless, the findings support the promise of body imaging at field strengths up to 14 T. The computed uiSNR maps may serve as performance benchmarks for receive arrays.

## Funding

This work was supported by HORIZON EUROPE Framework Programme (101078393/MRITwins).

## Supporting information


**Data S1:** mrm70202‐sup‐0001‐Supinfo.docx.

## Data Availability

The uiSNR maps generated from the Ella and truncated Duke models are available at: https://github.com/Yu‐tingW/torso_uisnr.
