# Supplementary material for: Ultimate Intrinsic SNR in the Torso of Realistic Body Models
Source: Magn Reson Med. 2025 Nov 26;95(4):2409–19. doi: 10.1002/mrm.70202 (PMC12850614; doi:10.1002/mrm.70202)
Supplement: Supplementary file 1 — Data S1: mrm70202‐sup‐0001‐Supinfo.docx. [file MRM-95-2409-s001.docx]

# Supplementary Information

## uiSNR calculation with a truncated body model and truncated dipole cloud

Compared to uiSNR simulations with the complete body model, simulations using truncated body models differ in two key aspects. First, the truncated body model introduces different boundary conditions compared to the complete model. Second, the dipole cloud applied in the truncated model constitutes only a subset of the dipole cloud used in the full simulations with complete coverage. To assess the potential effects of these differences on the uiSNR simulation results, we conducted several additional simulations using the Ella body model. The following simulations were performed at $B_{0}$ = 7 T: (1) the complete Ella model covered by a fully surrounding dipole cloud, (2) the complete Ella model partially covered by a truncated dipole cloud circumferentially around the torso part, and (3) the Ella model truncated from the neck to the proximal third of the femur, covered by the same truncated dipole cloud as in case 2. The simulation setups are illustrated in Supplementary Figure 1. In all cases, a basis set containing 3000 basis vectors was generated and used for uiSNR calculation.


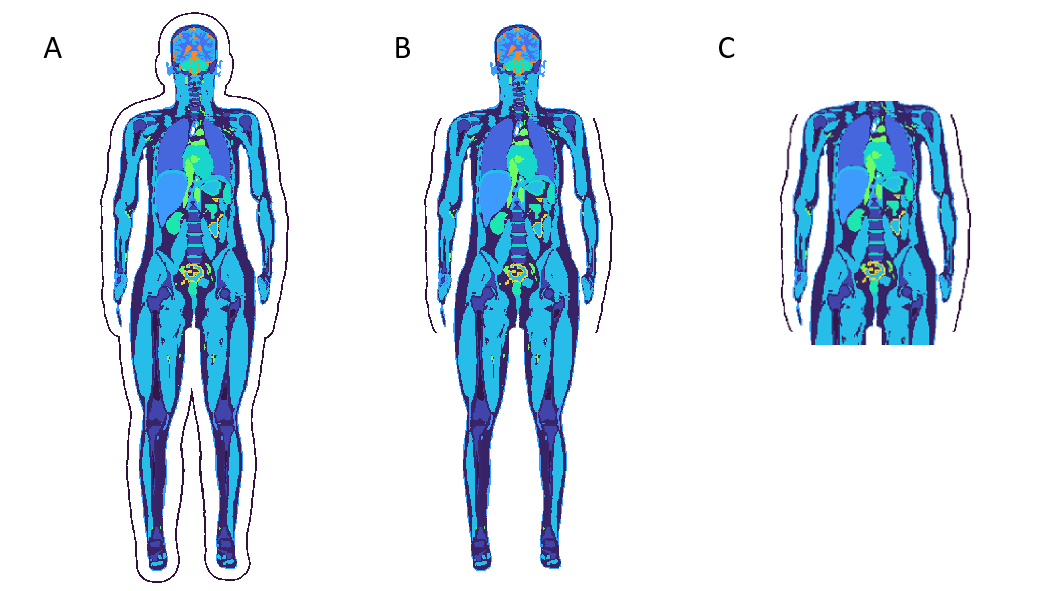


Supplementary Figure 1 Simulation setups. (A) Case 1: the complete Ella model covered by a fully surrounding dipole cloud. (B) Case 2: the complete Ella model partially covered by a truncated dipole cloud. (C) Case 3: the truncated Ella model covered by a truncated dipole cloud.

The relative ratios between the uiSNR maps obtained in these cases are shown in Supplementary Figure 2. It can be observed that, within the torso region of interest where convergence is considered to be achieved, the relative ratios between these three cases are close to 1. Interestingly, applying a partially covering dipole cloud to the complete body model yields higher SNR in peripheral regions with the same number of basis functions. This suggests that more localized dipole clouds may be more efficient in exciting peripheral locations, while in the region satisfying the 1% criterion, the deviation is generally negligible, being near-zero in the center of the body model and slightly increasing toward the border of the convergence mask. It is also interesting to notice that a substantial proportion of uiSNR can still be achieved in the head even when the dipole cloud does not directly cover the head. When comparing the complete model and truncated model using the same truncated dipole cloud, differences mainly appear near the truncation cross sections. As these regions are mostly located outside the convergence boundary of the truncated model, the truncated model yields result comparable to the complete model when the analysis is limited to the region with convergence.


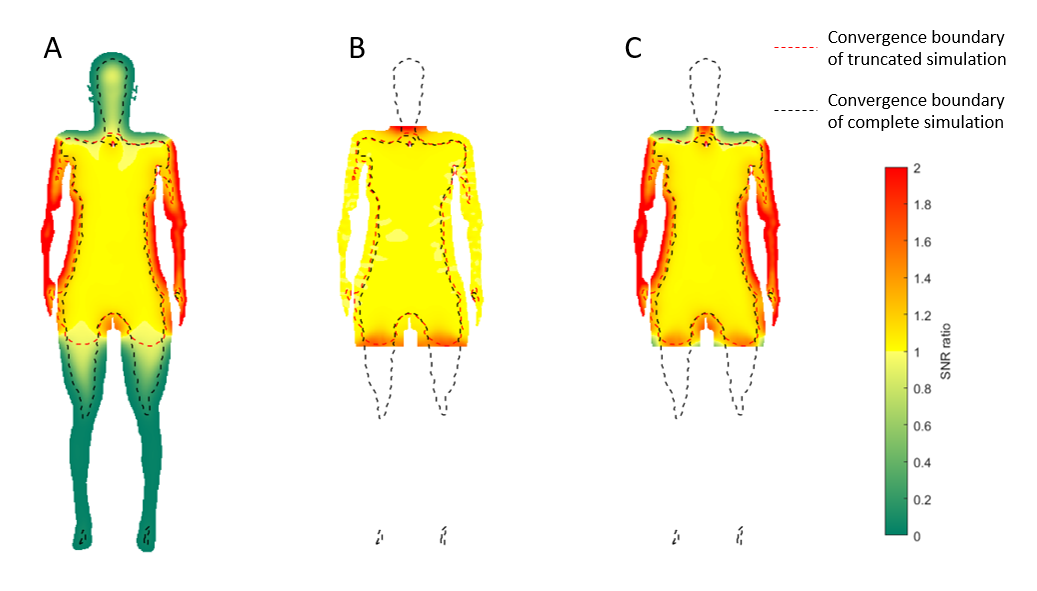


Supplementary Figure 2 SNR ratio between the cases. (A) Ratio between case 2 and case 1. (B) Ratio between case 3 and case 2. (C) Ratio between case 3 and case 1.

Considering both factors together, within the region with convergence defined by the 1% criterion, simulations based on a truncated model with a partially covering dipole cloud can yield comparable uiSNR values to those obtained using the complete body model. These results therefore support the validity of the simulations performed with the truncated models.

## uiSNR scaling for common X-nuclei

Supplementary Table 1 Field ranges of linear and superlinear uiSNR increase for several nuclei.

| Atomic nucleus | $\frac{\gamma}{2 \pi}$ (MHz/T) | Linear increase field range (T) | Superlinear increase field range (T) |
| --- | --- | --- | --- |
| ^1^H | 42.58 | 0.55 – 3 | 5 – 14 |
| ^2^H | 6.54 | 3.6 – 19.5 | 32.6 – 91.1 |
| ^13^C | 10.71 | 2.2 – 11.9 | 19.9 – 55.7 |
| ^15^N | -4.32 | 5.4 – 29.6 | 49.3 – 138.0 |
| ^17^O | -5.77 | 4.1 – 22.1 | 36.9 – 103.3 |
| ^19^F | 40.08 | 0.6 – 3.2 | 5.3 – 14.9 |
| ^23^Na | 11.26 | 2.1 – 11.3 | 18.9 – 52.9 |
| ^31^P | 17.24 | 1.4 – 7.4 | 12.3 – 34.6 |
| ^35^Cl | 4.21 | 5.6 – 30.3 | 50.6 – 141.6 |
| ^39^K | 1.99 | 11.8 – 64.2 | 107.0 – 299.6 |
